# The Influence of Chain Microstructure of Biodegradable Copolyesters Obtained with Low-Toxic Zirconium Initiator to *In Vitro* Biocompatibility

**DOI:** 10.1155/2013/176946

**Published:** 2013-08-25

**Authors:** Arkadiusz Orchel, Katarzyna Jelonek, Janusz Kasperczyk, Piotr Dobrzynski, Andrzej Marcinkowski, Elzbieta Pamula, Joanna Orchel, Ireneusz Bielecki, Anna Kulczycka

**Affiliations:** ^1^Department of Biopharmacy, School of Pharmacy, Medical University of Silesia, Narcyzów 1, 41-200 Sosnowiec, Poland; ^2^Centre of Polymer and Carbon Materials, Polish Academy of Sciences, M. Curie Sklodowskiej 34, 41-819 Zabrze, Poland; ^3^Department of Biomaterials, AGH University of Science and Technology, Mickiewicza 30, 30-059 Krakow, Poland; ^4^Department of Molecular Biology, Medical University of Silesia, Narcyzów 1, 41-200 Sosnowiec, Poland; ^5^Department of Pediatric Surgery, Medical University of Silesia, Medyków 16, 40-752 Katowice, Poland

## Abstract

Because of the wide use of biodegradable materials in tissue engineering, it is necessary to obtain biocompatible polymers with different mechanical and physical properties as well as degradation ratio. Novel co- and terpolymers of various composition and chain microstructure have been developed and applied for cell culture. The aim of this study was to evaluate the adhesion and proliferation of human chondrocytes to four biodegradable copolymers: lactide-coglycolide, lactide-co-*ε*-caprolactone, lactide-co-trimethylene carbonate, glycolide-co-*ε*-caprolactone, and one terpolymer glycolide-colactide-co-*ε*-caprolactone synthesized with the use of zirconium acetylacetonate as a nontoxic initiator. Chain microstructure of the copolymers was analyzed by means of ^1^H and ^13^C NMR spectroscopy and surface properties by AFM technique. Cell adhesion and proliferation were determined by CyQUANT Cell Proliferation Assay Kit. After 4 h the chondrocyte adhesion on the surface of studied materials was comparable to standard TCPS. Cell proliferation occurred on all the substrates; however, among the studied polymers poly(L-lactide-coglycolide) 85 : 15 that characterized the most blocky structure best supported cell growth. Chondrocytes retained the cell membrane integrity evaluated by the LDH release assay. As can be summarized from the results of the study, all the studied polymers are well tolerated by the cells that make them appropriate for human chondrocytes growth.

## 1. Introduction

 Biodegradable polymers such as aliphatic polyesters are widely used in medicine and pharmacy. Because of their biocompatibility, they are commonly utilized to produce surgical fibers and devices for bone fracture internal fixation [1, 2]. They can be also useful for tissue engineering and controlled drug delivery [2, 3]. Homo- and copolymers containing glycolidyl, lactidyl, caproyl, and carbonate units belong to the most frequently used biomaterials [2, 4]. These polymers are generally well tolerated by organism as well as their degradation products, which are eliminated from body by normal metabolic pathways [4, 5]. Lactic acid and glycolic acid are the degradation products of polylactide and polyglycolide, respectively. They have been shown to be well tolerated by cells *in vitro*; however, their impact on cell proliferation was dependent on the cell type. Generally, inhibition of cell growth observed at very high concentrations of lactic acid and glycolic acid was attributed by the authors to the increased osmolality of culture medium [6–8]. Cells of various types, including fibroblasts, chondrocytes, osteoblasts, smooth muscle cells, endothelial, cells and keratinocytes attached and proliferated on the surface of aliphatic polyesters of various composition. However, some reduction in cell growth has often been reported [6, 7, 9–13]. Other studies have demonstrated rewarding biocompatibility characteristics of poly(ester carbonate)s based on poly(1,3-trimethylene carbonate) [14, 15]. Copolymers containing TMC units are very useful for many biomedical applications because of their increased flexibility, the reduced acidity of the degradation products, surface erosion, and the distinctive profile of hydrolytic degradation [16]. 

Nevertheless, biocompatibility of aliphatic polyesters is controversial. Extracts containing degradation products of polylactide and polyglycolide exerted strong cytotoxic effect on mouse fibroblasts *in vitro* [17, 18]. In some laboratory and clinical studies with relatively long follow-up times bad tissue response and adverse effects were observed. The most common problems were nonspecific foreign body reaction and bone resorption [19, 20]. In mouse intraperitoneal injection of polylactide particulates induced inflammatory response and foreign body formation [21]. It has also been demonstrated that long-term implants of PLLA and PCLLA induced formation of tumors in rats [22, 23]. These adverse effects were attributed by authors to the breakdown of the polymers and the accumulation of degradation products. In our opinion, they also could result from the presence of residual catalysts and initiators remaining in the product after polymerization.

The most often used initiators in the production process of biodegradable aliphatic polyesters employed in medicine are tin compounds [24–26]. Because of the known high toxicity of tin compounds, these polymers cannot be considered as completely biocompatible. Complete elimination of tin compounds from the synthesized materials is practically impossible, which results in their slow penetration into patient's blood circulation system [27]. The level of residual tin can reach values of 100–530 ppm in both, commercially available and synthesized in the laboratory scale polylactides [28, 29]. It is known that tin compounds even in trace amounts are especially dangerous for small children and endanger proper functioning of sensitive tissues (e.g., brain tissue) [30]. Chang [31] reported that trimethyltin-treated neonatal rats appeared to be significantly stunned in growth. Webber et al. [32] found out that growth-plate chondrocytes were more sensitive to the cytotoxic effects of the alkyltins than were articular chondrocytes. The stannous ion can generate reactive oxygen species capable to damage cellular DNA [33]. Because of these facts, several attempts were undertaken to synthesize biodegradable polymers with the use of initiators more compatible with human organism as lithium, magnesium, iron, zinc, or zirconium compounds [29, 34–36]. Zirconium compounds were found to be tens times less toxic than analogous tin compounds. Drugs and cosmetics containing zirconium compounds are admitted by FDA (Food and Drug Administration) for use. Zirconium acetylacetonate was successfully used as an initiator of polymerization and allowed to obtain copolymers of glycolide, lactide, *ε*-caprolactone, or trimethylene carbonate (TMC) with enough high molecular masses to be used in production of surgical materials [34, 37]. Dobrzynski et al. [34] compared the microstructure and properties of copolymers of glycolide with L-lactide synthesized by use of zirconium acetylacetonate and analogous copolymers obtained under the same conditions in copolymerization initiated by Sn(oct)_2_. Copolymers synthesized with Zr(acac)_4_ displayed more segmental structure as a consequence of a lower rate of transesterification in the copolymerization process. They had higher crystallinity, better mechanical properties, and higher resistance for thermal degradation. 

In the presented study the usefulness of five different polymers for cartilage engineering was analyzed. The limited capacity of articular cartilage to regenerate significantly impedes treatment of damaged cartilage. Therefore, the clinical need for effective method of therapy of cartilage defects is the important reason of the rapid development of tissue engineering techniques [38]. Tissue engineering is an interdisciplinary scientific field which aims at the development of biological substitutes that restore, maintain, or improve the function of damaged tissues [39]. In order to create a new tissue the respective cells have to be seeded on a three-dimensional scaffold providing the necessary support for cells to proliferate and maintain their differentiated functions [40]. The choice of scaffold material has a crucial importance for the development of engineered tissue. Scaffold material should be biodegradable and its degradation products must be nontoxic and able to get metabolized and cleared from the body. Biodegradable synthetic polymers as aliphatic polyesters seem to be the most promising materials for tissue engineering applications. Their mechanical properties and degradation rate can be controlled in a wide range by modification of polymer composition and microstructure of the chains [34, 40]. Surface characteristics of a biomaterial determine the kind of adsorbed biological molecules. The properties of the molecules on the surface will exert strong impact on processes of the recruitment, attachment, proliferation, and differentiation of cells. Therefore, the physicochemical characteristics of the polymer surface layer, such as wettability, surface free energy, electrical charge, crystallinity, topography, and presence of certain atoms or chemical functional groups, for example, carbon, amine groups, or oxygen groups determine phenotypic properties of cultured cells [41, 42]. 

The aim of this study was to characterize the polymer chain microstructure and surface properties of a set of five aliphatic polyesters recently synthesized with the use of zirconium acetylacetonate and to evaluate the affinity of human chondrocytes to these materials. Selection of the biodegradable polyesters materials for chondrocytes culture was done on the basis of degradation process investigations presented in our previous papers [36, 43–48].

## 2. Materials and Methods

### 2.1. Synthesis of Copolymers

Five kinds of polymers were used to prepare substrates for chondrocytes culture: (1) poly(L-lactide-coglycolide) PLAGA 85 : 15; (2) poly(L-lactide-co-*ε*-caprolactone) (PLACL) 75 : 25; (3) poly(L-lactide-co-trimethylene carbonate) (PLATMC) 72 : 28; (4) poly(glycolide-co-*ε*-caprolactone) (PGACL) 8 : 92; (5) poly(L-lactide-co-*ε*-caprolactone-coglycolide) (PLACLGA) 66 : 24 : 10. Copolymers were synthesized according to the method described previously [16, 34, 43, 44]. Briefly, the syntheses were performed in bulk by ring opening polymerization (ROP) of lactones: glycolide, L-lactide, *ε*-caprolactone (Purac), TMC (Boehringer Ingelheim) with vacuum line for degassing and sealing the ampoules, using zirconium (IV) acetylacetonate (Zr(acac)_4_) (Aldrich) as a nontoxic initiator. In the case of PGACL 8 : 92; (PLATMC) 72 : 28, and (PLACLGA) 66 : 24 : 10 copolymers the copolymerization reaction was conducted at 110°C with an I/M molar ratio of 1/800. PLAGA 85 : 15 and PLACL 75 : 25 copolymers were synthesized at 120°C with an I/M molar ratio of 1/1000. The obtained materials were precipitated with methyl alcohol to remove unreacted monomers and then dried at 50°C under vacuum. 

### 2.2. Characteristic of the Studied Copolymers

Copolymer microstructure was characterized by means of the parameters determined from ^1^H NMR and ^13^C NMR spectra according to the equations presented in the literature: the percentage content of lactide (F_LL_), glycolide (F_GG_), carbonate (F_c_), and caproyl (F_Cap_) units, the average length of the lactide (*l*_LL_), glycolide (*l*_GG_), carbonate (*l*_c_), caproyl (*l*_Cap_) blocks, transesterification of the second mode (T_II_), and randomization ratio (*R*). The ^1^H NMR spectra of the studied copolymers were recorded at 600 MHz and ^13^C NMR at 125 MHz with AVANCE II Ultra Shield Plus Bruker 600 MHz spectrometer and a 5 mm sample tube. CDCl_3_ or DMSO-d_6_ was used as a solvent. The spectra were obtained at 28°C with 32 scans, 3.74 s acquisition time and 7 *μ*s pulse width in the case of proton spectra, and 30000 scans, 1.8 s acquisition time, 9 *μ*s pulse width and 3 s of delay time between pulses in the case of carbon spectra.

The molecular weights and molecular weight distribution coefficients of the obtained copolymers were determined by gel permeation chromatography with a Physics SP 8800 chromatograph (tetrahydrofuran was used as the eluant, the flow rate was 1 mL/min, and Styragel columns and Shodex SE 61 detector were used). The molecular weights were calibrated with polystyrene standards.

Glass transition temperature was examined by differential scanning calorimetry (DSC) with a DuPont 1090B apparatus calibrated with gallium and indium. The samples were scanned from about −20°C to 220°C at a heating rate of 20°C/min and then quenched into liquid nitrogen.

The contact angles were measured by the sessile drop method using an automatic drop shape analysis system DSA 10 Mk2 (Kruss, Germany).

### 2.3. Preparation of Polymeric Substrates for Human Chondrocytes Culture

The obtained copolymers were dissolved in 1,1,1,3,3,3-HFIP (Fluka) to obtain polymer solutions with the same viscosity. The solutions were subsequently used to fabricate the polymeric films covering the bottoms of 96-well plates (Corning). To ensure the complete evaporation of the solvent the films were air dried for seven days and subsequently vacuum dried for another week. The films were sterilized with exposure to *γ*-irradiation (Co-60 (2,5 MRad)). 

### 2.4. AFM Measurements

The surface morphology of the studied polymeric matrices was analyzed by means of AFM technique, with the use of a multimode AFM instrument (MultiMode, di-Veeco, USA, CA) with NanoScope 3D, which was operating in the tapping mode in air with standard 125 *μ*m single-crystal silicon cantilevers (Model TESP; di-Veeco, USA, CA). The piezoelectric scanner had a scan range ~ 10 × 10 *μ*m. All samples were imaged at room temperature. 

The matrices were prepared by solution of each kind of copolymer in 1,1,1,3,3,3-HFIP (Fluka). The solution was cast on a metal plates of 1 cm diameter and evaporated at ambient temperature. Than, the films were dried under reduced pressure. The software package WS × M (Nanotec Electronica) was used for image processing [49].

### 2.5. Human Chondrocytes Culture

Chondrocytes were isolated from the specimen of cartilage from the nasal septum of thirty-three-years-old patient. Harvested cartilage was washed with the chilled balanced salt solution, diced, and incubated overnight at 37°C in complete medium with 0.2% collagenase (ICN) and 0.1% hyaluronidase (Sigma). Complete medium contained MEM (Minimum Essential Medium, Sigma) supplemented with 10% fetal bovine serum (FBS, Invitrogen), 100 U/mL penicillin, 100 *μ*g/mL streptomycin (Sigma), 1 × MEM-Non Essential Amino Acids (Invitrogen), and 10 mM HEPES buffer (Applichem). The resulting cell suspension was filtered with a 100 *μ*m cell strainer (Falcon, Becton Dickinson) to remove any undigested tissue and rinsed of the enzymes solution with the growth medium. The chondrocytes were counted with a hemocytometer and viability of the cells was determined by the trypan blue exclusion test. Isolated cells were seeded in tissue culture flasks, at a density of 2 × 10^4^ cells/cm^2^, and cultured at 37°C in a humidified atmosphere containing 5% CO_2_. For the following experiments, cells at four passages were used.

### 2.6. Cell Adhesion and Proliferation

Prior to cell seeding the 96-well plates coated with the uniform thin polymer films were washed with HBSS. To study the cell adhesion, chondrocytes suspended in the complete growth medium were plated into 96-well plates at a density of 10^4^ cell/well. The cells were allowed to adhere to the substrates undisturbed in the incubator for 0.5, 1, and 4 hours. At the end of incubation, the medium was aspirated and the wells were washed with PBS to remove the residual culture medium and unattached cells. Subsequently the plates were frozen at −70°C. Adherent cell number was quantitated by means of CyQUANT Cell Proliferation Assay Kit (Molecular Probes) according to the manufacturer's instruction. The basis for this assay is the use of a green fluorescent dye (CyQUANT GR dye), which exhibits strong fluorescence enhancement when bound to cellular nucleic acids. Fluorescence emission was measured at a wavelength of 535 nm after excitation at 485 nm using a Victor 1420 Multilabel Counter (Perkin-Elmer Instruments). Sample fluorescence values were converted into cell numbers using a standard curve constructed by plotting CyQUANT GR fluorescence against cell counts.

For the assessment of the cell proliferation, cells were seeded into 96-well plates (2 × 10^3^ cells/well) coated with the polymers and cultured for 4 days. Cell number was quantitated using CyQUANT Cell Proliferation Assay Kit as described above.

### 2.7. Lactate Dehydrogenase (LDH) Assay

To assess the cell membrane integrity, LDH activity in the culture medium was measured. LDH is a stable cytosolic enzyme that is released to the culture medium upon cell damage. 200 *μ*L of the complete medium containing 5 × 10^3^ cells was transferred to each well in a 96-well plate. Cells were allowed to attach to the polymeric substrates for 24 h. Subsequently the complete culture medium was removed, the plates were washed with HBSS, and wells were filled with 200 *μ*L of fresh medium containing MEM, 2% FBS, 100 U/mL penicillin, 100 *μ*g/mL streptomycin, 1× MEM-nonessential amino acids and 10 mM HEPES buffer, 10 *μ*g/mL insulin, 5.5 *μ*g/mL transferrin, and 6.7 ng/mL sodium selenite. After the medium was changed, the cells were cultured for the next three days. LDH activity was determined both in the culture medium and in the cells with the use of “*In Vitro* Toxicology Assay Kit, Lactate Dehydrogenase Based” (Sigma) according to the manufacturer's instruction. The assay was performed in 96-well microliter plates. The absorbance was measured at wavelengths of 490 nm and 690 nm (background absorbance) using a microplate reader (MRX Revelation, Dynex Technologies). Subsequently, the percentage of liberated enzyme was calculated for chondrocytes growing on each polymeric material.

### 2.8. Statistical Analysis

The data were analyzed using a one-way ANOVA and *t*-test. All the results are expressed as means ± SD. *P* value of <0.05 was considered statistically significant.

## 3. Results and Discussion

The cell culture model is very useful to evaluate the biocompatibility of medical materials since under controlled *in vitro* conditions it is possible to investigate both direct cell interactions with the polymer surface and the impact of soluble factors released from the polymer on cell viability. The cells chosen for the assay should be representative of the cell population contacting with polymeric material after its implantation.

Because of the wide use of biodegradable materials in tissue engineering, it is necessary to obtain polymers with different mechanical and physical properties as well as degradation ratio. Therefore, novel co- and terpolymers of various composition and chain microstructure are currently developed and applied as scaffolds for cell culture.

The microstructure of the obtained copolyester is influenced by the kind of applied initiator as well as by the transesterification occurring during the synthesis. It was proved that it is possible to control to some extent the chain structure of the synthesized copolymer and at the same time its properties by adjusting the kind of initiator and the conditions of copolymerization [34]. It was determined that using Zr(acac)_4_ as initiator of copolymerization reaction of lactide and TMC [50], glycolide with *ε*-caprolactone [51], lactide with glycolide [34], or terpolymers of lactide, glycolide, and *ε*-caprolactone [52] allowed to obtain polymers with high molecular ratio. When higher temperature was applied, it is also possible to obtain more random copolymers as a result of transesterification of the second mode [52]. In case of copolymers with glycolide and/or lactide, transesterification of the first mode takes place during intermolecular exchange of lactidyl (LL) and/or glycolidyl (GG) units or their multiplets. Transesterification of the second mode causes bond cleavage in the lactidyl or glycolidyl units, forming lactyl (L) –OCH(CH_3_)CO– and glycolyl (G) –OCH_2_CO– sequences [52]. The transesterification results in the redistribution of the sequences along the polymer chains leading to changes in the chain structure and the lengths of microblocks [53]. 

 In the present study, five kinds of copolymers were synthesized and their biocompatibility with human chondrocytes was investigated. All of the studied copolymers characterized high molecular ratio appropriate for tissue engineering application. The analysis of the copolymer microstructure was performed by means of NMR spectroscopy. The molar ratio of comonomer, the average length of the lactidyl (*l*_LL_), glycolidyl (*l*_GG_), caproyl (*l*_Cap_), and carbonate (*l*_T_) unit, and value of randomization and transesterification of the second mode ratio were determined. Characteristic of the used copolymers is presented in Table 1. 

Two of them had multiblock structure: PLAGA 85 : 15 (*R* = 0.43) and PLATMC 72 : 28 (*R* = 0.56), while the rest of the polymers were random. Transesterification of the second mode was determined for all copolymers except from the PLACL 75 : 25. The CapLCap sequences that are formed during transesterification reaction were not detected in ^13^C NMR spectra of the PLACL 75 : 25. In case of PGACL 8 : 92 and PLATMC 72 : 28, the value of T_II_ was above 1, which means that longer alternative sequences are propagated in copolymers [35]. 

**Figure 1:**
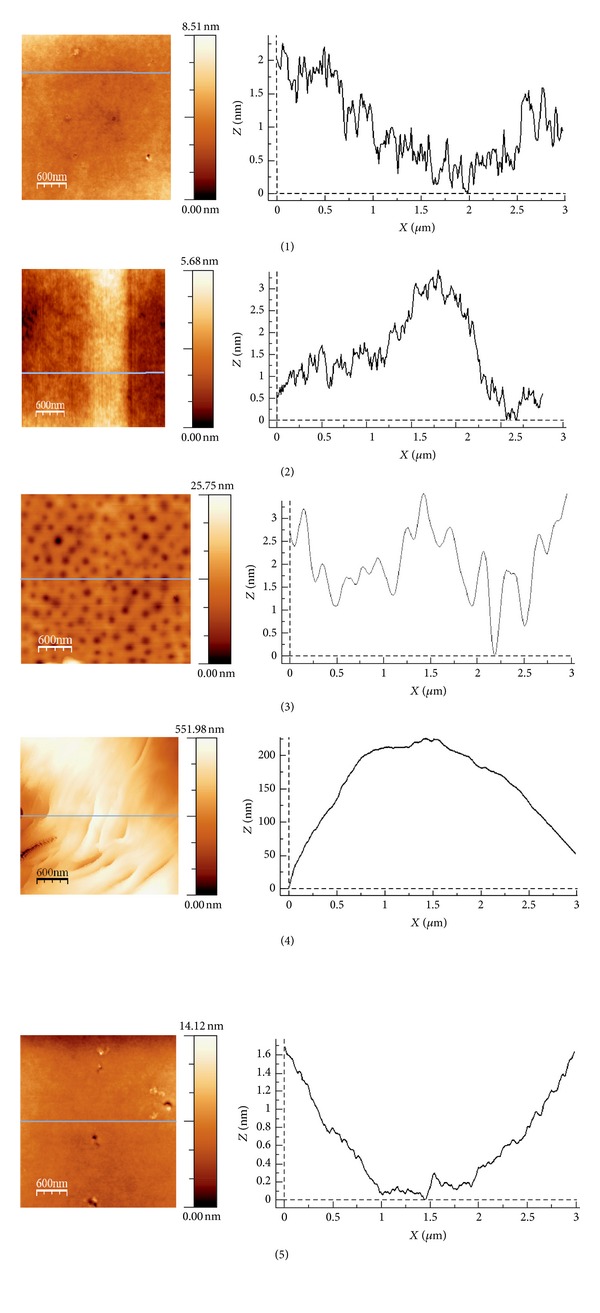
AFM topographic images of PLATMC (1), PGACL (2), and PLAGA (3), PLACL (4), PLACLGA (5).

 The solvent casting technique allowed to produce homogenously thin films with polymers described above. Polymer surface analysis at nanoscale with AFM revealed that films of PLAGA 85 : 15, PLACL 75 : 25, and PLACLGA 66 : 24 : 10: were extremely smooth (Figure 1). 

In contrast, wave-like surface perturbations up to 500 nm in height were observed on PGACL 8 : 92. On the surface of PLATMC 72 : 28 numerous nanopores less than 10 nm deep were visible (the mean number in the analyzed area 3 *μ*m × 3 *μ*m was 78). It is believed that the surface roughness and topography, together with the other factors as the surface chemistry and wettability, affect the cell behavior [54]. Process of the cell adhesion is rather complex and it is preceded by adhesion of extracellular matrix (ECM) molecules to the polymeric surface. Cells bind to specific amino acid sequences of these molecules through membrane receptors (e.g., integrins). A consequence of ligand binding is the formation of focal adhesion plaques that link integrins with a cytoskeleton and generate intracellular signals. Too rough surface can hamper the development of focal adhesion plaques and cell spreading [55]. Additionally, sharp irregularities may damage mechanically the cells. On the other hand, too smooth surfaces can also negatively affect the cell adhesion [54]. 

**Figure 2:**
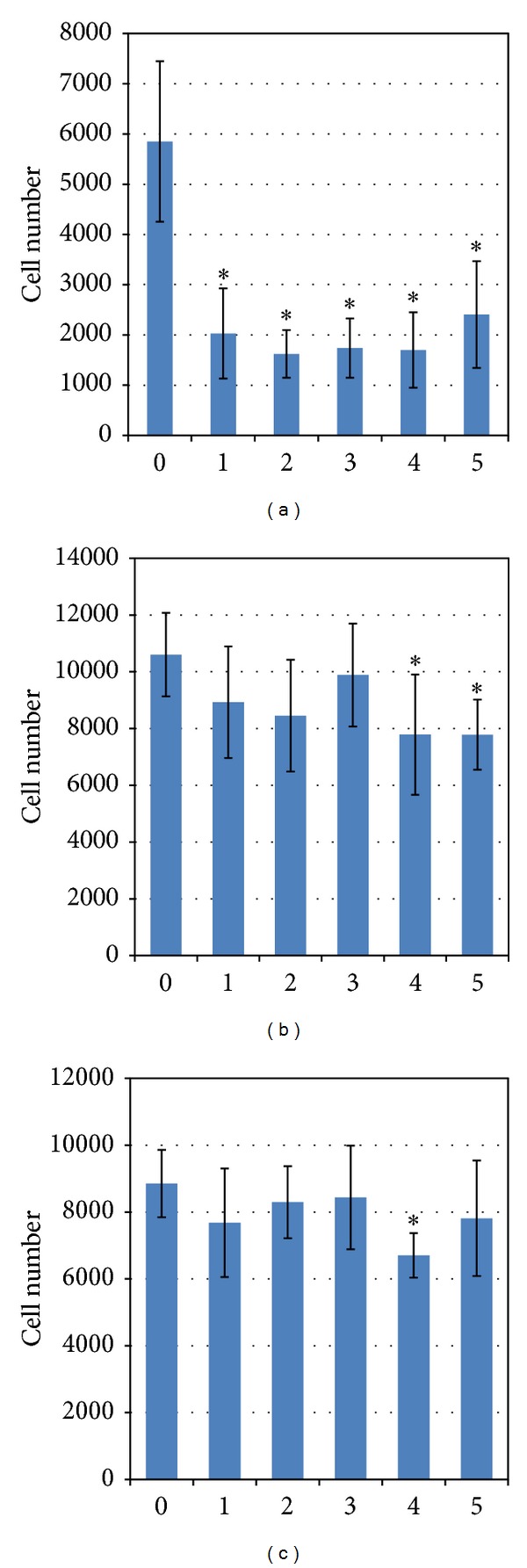
Number of human chondrocytes adhered to the various polymer films after 0.5 (a), 1 h (b), 4 h (c) of incubation. (0) Control—standard tissue culture polystyrene (TCPS); (1) PLAGA (2) PLACL (3) PLATMC (4) PGACL (5) PLACLGA; *P < 0.05 compared to control.

Fluorimetric assays of cell adhesion revealed that chondrocytes attached more slowly to polymeric films compared to control. As shown in Figure 2, after 0.5 h of incubation much lower amount of attached cells was observed on all polymeric materials than on standard tissue culture polystyrene (TCPS). However, the number of adherent chondrocytes continued to increase over time and after 4 h of incubation high number of cells, comparable to that on the control TCPS, was seen on the surface of the majority of the studied materials (Figures 2(b) and 2(c)). At the last data point, significantly less cells were found adhered solely on the PGACL film.

**Figure 3:**
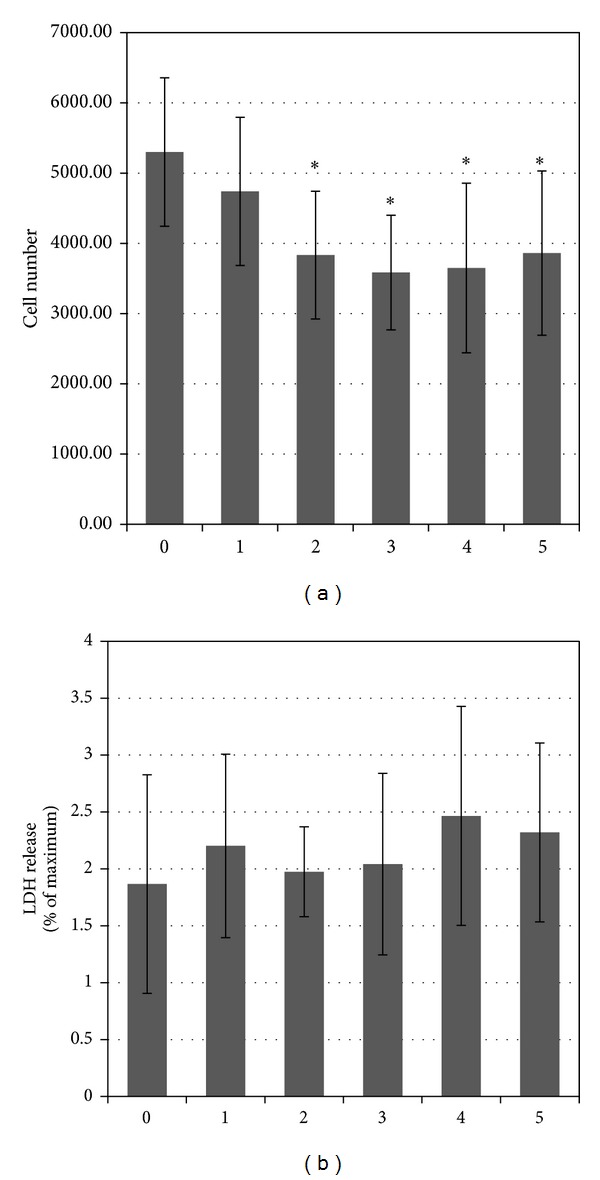
(a) Growth of chondrocytes cultured for 4 days on polymeric films. (b) Percentage of LDH released to medium. (0) Control—standard tissue culture polystyrene (TCPS); (1) PLAGA (2) PLACL (3) PLATMC (4) PGACL 5) PLACLGA; *P < 0.05 compared to control.

The increased chondrocyte numbers both on the polymeric foils and on control surface at the end of the studied growth period (4 days) confirmed that proliferation occurred on all the substrates (Figure 3(a)). 

**Figure 4:**
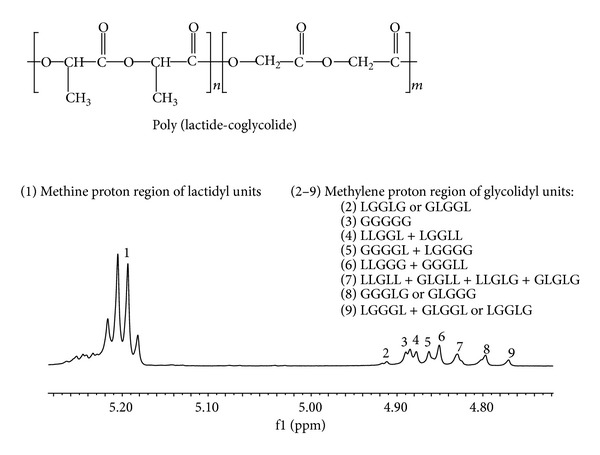
^1^H NMR (600 MHz) spectrum of PLAGA 85 : 15 recorded in DMSO-d_6_. Methine proton region of lactidyl unit (1) and methylene proton region of glycolidyl unit (2–9).

Generally, chondrocyte number on TCPS was significantly higher than those on the polymeric films. Among the studied materials PLAGA best supported cell growth. The lactide content as well as the average length of the lactidyl blocks were the highest in comparison with the rest of the studied polymers (Table 1). This copolymer characterized also the most blocky structure. The ^1^H NMR spectrum of PLAGA 85 : 15 is presented in Figure 4. 

**Figure 5:**
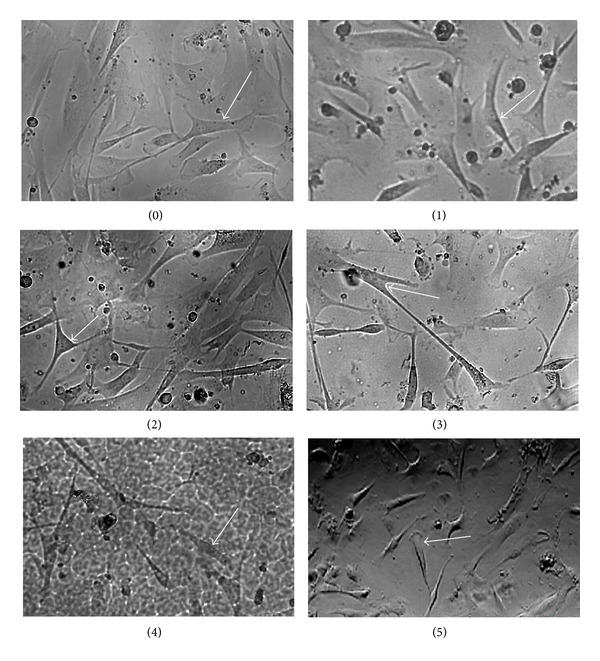
Morphology of chondrocytes cultured on the (0) standard tissue culture polystyrene (TCPS) and polymeric films: (1) PLAGA (2) PLACL (3) PLATMC (4) PGACL (5) PLACLGA, evaluation under phase contrast microscopy.

Phase contrast microscopy observations of the chondrocytes revealed that they adhered and spread on the surface of both TCPS and all the materials studied. They displayed a bipolar or multipolar morphology typical of chondrocytes in two-dimensional monolayer cultures (Figure 5).

To examine whether plasma membrane integrity was affected by studied materials, LDH release was evaluated. The leakage of LDH into culture medium provides a reliable and sensitive marker of cellular cytotoxicity in biocompatibility studies. The amount of cell death was expressed as the percentage of released LDH per total LDH on each substrate and the results are presented in Figure 3(b). It can be concluded that cell viability did not appear to be affected by the kind of the substrate.

**Figure 6:**
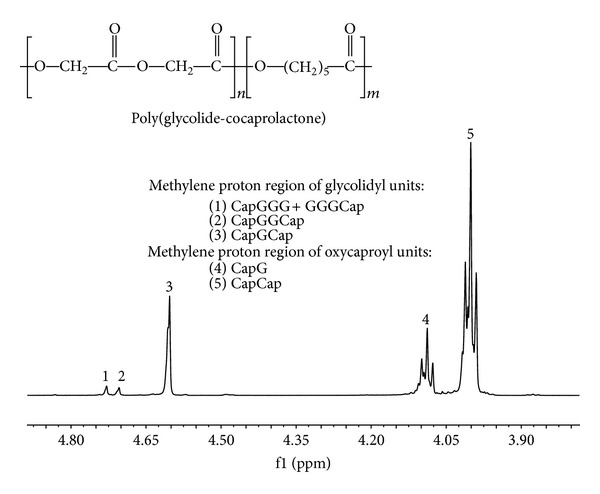
^1^H NMR (600 MHz) spectrum of PGACL 8 : 92 recorded in CDCl_3_. Methylene proton region of glycolide units (1–3) and methylene proton region of *ε*-oxycaproyl unit (4-5).

It is worth noting that polymer water contact angle did not correlate with degree of chondrocyte attachment and proliferation. It was probably a consequence of relatively small range of the contact angle values. Differences in hydrophilicity of the polymers could be too small to elicit a differential cell response. The lack of a correlation between the chondrocyte attachment and water contact angle was previously observed by Ishaug-Riley et al. [11] These authors reported relatively low colonization of substrates with a high molar ratio of *ε*-caprolactone. In other studies very weak growth of L929 mouse fibroblasts [41] and osteoblast-like MG63 [56] cells on PGACL 10 : 90 films was found. Poor cell affinity of *ε*-caprolactone based materials was attributed by the authors to their crystalline structure, rough surface, low molar percentage of oxygen at the surface, plasticity, hydrophobicity, and low surface free energy [41]. These could also be the reason of slower adhesion of chondrocytes on PGACL 8 : 92 was somewhat compared to the other polymers observed in our study. Moreover, as was mentioned before wave-like surface perturbations on the surface of this material were revealed in AFM analysis (Figure 1). Importantly, contrary to blocky PLAGA 85 : 15, this copolymer characterized high transesterification of the second mode and randomization ratio (Table 1). The ^1^H NMR spectrum of PGACL 8 : 92 is presented in Figure 6. The resonance lines in PGACL spectrum were assigned according to the literature [57].

Nevertheless, the cell proliferation rate on PGACL 8 : 92 was similar to that seen on the other polymers with the exception of PLAGA 85 : 15. Tang et al. clearly demonstrated the impact of the solvent used on the surface properties of solvent cast PCL films [13]. PCL has relatively numerous nonpolar methyl groups along its backbone, but the kind of solvent determines conformation of polymer molecules and arrangement of polar and nonpolar groups at the material surface. The use in our study of a highly polar solvent (HFIP) had to favour the exposition of the hydrophilic functional groups at the polymer surface. It could increase adhesion of the extracellular matrix proteins to the polymeric surface and thereby stimulate cell proliferation. Theoretically, poor cell adhesion and growth of cells on resorbable polymers might be caused by their hydrolytic degradation and release of soluble acidic products accumulating in the culture medium and exerting negative effect on cell viability. However, this supposition must be ruled out, because LDH test showed no significant difference between the cell viability for all the test groups. Moreover, materials tested in the present study are not very susceptible to hydrolytic degradation and the cell incubation period was relatively short (96 h). The degradation process of these polymers was studied in detail by our team and the majority of the results were published elsewhere [43–48]. Generally, it could be summarized that polymer degradation started just after immersing in PBS and reaction proceeded gradually through the next several weeks. 

## 4. Conclusions

The *in vitro* biocompatibility and the influence of copolymer chain microstructure on chondrocyte adhesion and proliferation was tested. It was found that copolymers synthesized with Zr(acac)_4_ displayed more segmental structure compared to commonly used PLAGA initiated by Sn(oct)_2_ [34]. However, in our study PLGA 85 : 15, that characterized the most segmental structure among the studied polymers, provided the best support for cell growth. Although chondrocyte adhesion on the surface of studied materials was relatively slow after 0.5 h, compared to standard TCPS, the cells finally spread on the polymeric foils and proliferated. Only in case of PGACL 8 : 92, that characterized the highest randomization ratio among all analyzed polymers, adhesion proceeded a bit slower—the significant increase of cell number was observed after 4 h. It could have been caused by surface of this copolymer, which characterized wave-like perturbations. The rest of the studies materials had smooth (PLAGA 85 : 15, PLACL 75 : 25, and PLACLGA 66 : 24 : 10) or smooth with nanopores (PLATMC 72 : 28) surface. Chondrocytes retained the cell membrane integrity evaluated by the LDH release assay. Generally, it can be concluded that the studied polymers are well tolerated by the cells and appropriate for human chondrocytes growth, so they can be used in cartilage tissue engineering. The new methods of therapy of cartilage defects are especially demanded due to the limited capacity of articular tissue to regenerate. Therefore, in the next step the polymers synthesized with the use of low-toxic zirconium initiator should be used to prepare three-dimensional scaffolds to confirm their usefulness for chondrocytes growth. 

## Figures and Tables

**Table 1:** Characteristics of copolymeric chain microstructure.

No	Kind of copolymer/molar ratio	*M*_*n*_ (kDa)	*D*	*T*_*g*_ (°C)	The average length of the blocks	*R*	T_II_	*θ* [°]
1	poly(L-lactide-coglycolide) PLAGA/85 : 15	42.1	2.8	58	*l*_LL_ = 11.12 *l*_GG_ = 2.02	0.43	0.1	72.9 ± 2.6
2	poly(L-lactide-co-*ε*-caprolactone) (PLACL)/75 : 25	63.5	2.5	35	*l*_LL_ = 4.29 *l*_Cap_ = 1.59	0.75	—	87.1 ± 4.5
3	poly(L-lactide-cotrimethylene carbonate) (PLATMC)/72 : 28	36.0	2.0	42	*l*_LL_ = 5.57 *l*_T_ = 2.13	0.56	4.16	76.8 ± 0.9
4	poly(glycolide-co-*ε*-caprolactone) (PGACL)/8 : 92	40.0	2.0	−46	*l*_GG_ = 1.1 *l*_Cap_ = 4.94	1.1	1.15	78.7 ± 2.2
5	poly(L-lactide-coglycolide-co-*ε*-caprolactone) (PLACLGA)/66 : 24 : 10	50.0	1.9	29	*l*_LL_ = 5.25 *l*_GG_ = 0.94 *l*_Cap_ = 5.9	—	0.02	79.1 ± 1.4

*M*_*n*_: number-average molecular mass.

*D*: molecular weight distribution.

*T*_*g*_: glass transition temperature.

*l*_LL_, *l*_Cap_, *l*_GG_, *l*_T_: the average length of lactidyl, caproyl, glycolidyl, and carbonate sequences, respectively.

*R*: randomization ratio.

T_II_: transesterification of the second mode ratio.

*θ*: water contact angle.
